# In defense of quantitative metrics in researcher assessments

**DOI:** 10.1371/journal.pbio.3002408

**Published:** 2023-12-04

**Authors:** John P. A. Ioannidis, Zacharias Maniadis

**Affiliations:** 1 Departments of Medicine, of Epidemiology and Population Health, of Biomedical Data Science, and of Statistics, and Meta-Research Innovation Center at Stanford (METRICS), Stanford University, Stanford, California, United States of America; 2 SInnoPSis (Science and Innovation Policy and Studies) Unit, Department of Economics, University of Cyprus, Nicosia, Cyprus; 3 Department of Economics, University of Southampton, Southampton, United Kingdom

## Abstract

Using quantitative metrics to assess researchers is often seen as a poor choice compared with using qualitative assessments. In this Perspective, the authors argue in favor of using rigorous, field-adjusted, centralized, quantitative metrics in a bid to help improve research practices as a low-cost public good.

## Teaser

Qualitative assessments of researchers are resource-intensive, untenable in nonmeritocratic settings, and error-prone. Although often derided, quantitative metrics could help improve research practices if they are rigorous, field-adjusted, and centralized.

Scientists are continuously assessed for hiring, promotion, funding, and recognitions. A welcome movement for incentive reforms is aiming to align assessments with good scientific practice (e.g., open science, reproducibility, and diverse types of social impact). Measuring such progressive contributions and impact requires qualitative assessments that capture a wide range of achievements, beyond what traditional bibliometric quantitative indicators capture. Meanwhile, several influential international proposals for reforming research assessment have lambasted flawed quantitative metrics. For example, the San Francisco Declaration on Research Assessment (DORA) justifiably urges the abandoning of Journal Impact Factors (JIFs), and the thoughtful Leiden manifesto [1] offers skeptical advice against bibliometrics acquiring inordinate influence.

Quantitative metrics undoubtedly have limitations; however, their uncritical dismissal may aggravate injustices and inequities, especially in nonmeritocratic environments. We would argue that centralized, quantitative resources can serve as a public good at little or no marginal cost in diverse situations and settings. Their judicious use may even reduce manipulative practices in scientific publishing and lead to fairer allocation of credit.

In institutions with sufficient resources, the most important assessment for recruitment or for tenure is typically made by numerous highly respected assessors, including those external to an institution. Under ideal conditions, established, knowledgeable, responsible, and accountable peers will carefully review a candidate’s research production and wider impact. Faculty and researchers selected through such a process may excel in the broadest possible sense. Yet even so, one cannot always be sure that those chosen are the very best among the dozens or even hundreds of competing applicants. No assessment can perfectly rank very strong alternative candidates. Local needs and preferences, and strong elements of subjectivity, may decide who is recruited among several outstanding options. The same applies to very selective, competitive awards and recognitions.

For most institutions, choosing the best using qualitative assessment alone is almost impossible. Local faculty may lack sufficient expertise and experience and therefore be less capable judges of scientific quality, talent, or potential, while highly competent external reviewers can be notoriously difficult to entice to contribute to an evaluation. Furthermore, individuals with major contributions can be unwelcome in environments where mediocrity and/or corruption thrive. Corruption is difficult to measure, but it is probably highly prevalent worldwide [2]; like any societal structure, research environments may also be affected. In addition, many institutions may be at a loss as to how to appraise impact in a meaningful way. The influential Agreement on Reforming Research Assessment notes that qualitative metrics require additional resources from institutions. Most institutions, even if not affected by corruption, lack resources even for vital aspects of their operation and mission. Adding another level of local assessment bureaucracy will not help these institutions or research at large, and peer judgement may flounder in low-trust environments.

Even if qualitative assessments could be scaled and improved, researchers’ time is a scarce resource that already has excessive demands on it for performing all sorts of assessments. The journal peer review process already overspends that scarce resource to vet millions of paper submissions annually [3]. Greater emphasis on qualitative assessment of researchers raises further demands towards reviewing peers, this time not just their single papers, but entire corpora and CVs that are becoming increasingly inflated under “publish or perish” pressure. Under these circumstances, well-intentioned proposals urging individual institutions to dismiss quantitative research metrics seem misplaced. Instead of dismissing quantitative metrics, more emphasis should be placed on empowering resource-poor institutions via better metrics. An underappreciated feature of quantitative metrics that renders them a superior investment for society is that the marginal cost of using such metrics, once developed centrally (including information for all scientists and for all institutions), is very low.

A very simple economic framework can be used to analyze the problem. For research institutions, high-level qualitative judgment is a high-cost option. It is a private good because it does not yield a generalized framework or formula that can be used at low cost by others. Standardized quantitative metrics, conversely, have clear limitations, but they can become a public good, employed at almost zero cost by institutions not involved in their development. Resource-wealthy institutions may still prefer to use the expensive “private good,” while most institutions may opt for the “public good,” if available at almost zero cost. Exhorting resource-poor institutions to employ the unaffordable “private good” is counterproductive. Instead, we should strengthen the “public good,” namely, standardized metrics that cover all institutions and all scientists.

However, quantitative metrics are not without their flaws, and critics often lament their gaming potential. By Goodheart’s law, when metrics acquire power, they will be gamed [4]. Metrics such as counting the number of publications or derivatives thereof (e.g., counting JIFs’ sums, and to some extent, even the h-index) are indeed unreliable due to gaming. The most common gaming mechanism, gift (honorary) authorship to powerful scientists, appears across the board in scientific literature. It may happen even in journals considered to be the most prestigious and rigorous ones and may affect even some scientists perceived as leaders in their field. Thus, gift authorship may erode fair practices even at major scientific epicenters, while practices such as paper mills [5], massive self-citation [6], and citation farms [7] may create obscenely weird CVs, but mostly happen through journals with a limited impact on science. The optimal response to gaming is using metrics that quantify and detect extreme and spurious behaviors, such as hyperprolific authorship (especially with sudden accelerations upon acquiring administrative power), extreme self-citation, overdependence of a CV on massive coauthorship, and spurious orchestration of citations. This is currently feasible in large-scale, science-wide databases with standardized means and processes that can place researchers’ output in perspective against all other researchers in the same subfield worldwide; such resources are now freely and publicly available [8–10]. These efforts should be run centrally, instead of having each researcher, institution, and agency endlessly duplicating resources and efforts. Conversely, purely qualitative assessments of researcher impact cannot be meaningfully centralized/standardized and remain largely at the eye of the (local) beholder.

Besides citation gaming, some scientists also object that citations can be biased against gender, racial, or national groups; accrue slowly; and might not align with expert review. However, field-normalized measures can achieve parity with expert-provided peer review scores in interrater reliability [11]. Quantitative measures are also disinterested and largely consistent over time, unlike human judgement. Gender, racial, and country bias in metrics can be anticipated, probed, and corrected, while subjective human judgement suffers similar biases that are difficult to isolate and remove. Another caveat is the exclusion of journals from the global South from indexing databases, further disadvantaging researchers in these areas; however, this deficiency can be corrected with better inclusiveness.

Table 1 shows some desirable features of metrics as public goods. Bibliometrics should encompass indicators of best research practices (e.g., frequency of data sharing, code sharing, protocol registration, and replications) as a free, publicly available resource covering all the open-access literature [12]. Examples include PLOS’s Open Science Indicators, which are currently capturing data sharing in repositories, code sharing, and preprint posting, and the Dimensions Research Integrity product and its proposed Ripeta Score [13]. Centralized open-access assessments can also scrutinize for elements of poor research practices (e.g., signs of image manipulation) at a massive scale. For example, one science-wide assessment of top-cited scientists currently excludes from consideration those with retracted papers.

**Table 1:** Desirable features for quantitative metrics for research assessment.

Feature	Comments
Public good with low marginal cost	An increasing spectrum of bibliometric databases are available for free; commercial, subscription databases may also be used to generate freely accessible, publicly available indicators.
Science-wide, global coverage	Important to cover all scientists, so as to have the full comparative picture at a global level.
Transparent and reproducible	Open visibility allows verification, trust, and correction of any errors; methods of development and their rationale should also be documented.
Centralized	Having each candidate and each institution generate their own metrics causes confusion, lack of standardization, and unintentional or intentional errors.
Standardized	Proper adjustments (e.g., for scientific subfield) should be done in a rigorous way and should be uniform rather than be reinvented for each occasion (perhaps even with self-serving goals).
Reduced gaming potential	Some metrics are more difficult to game than others and should thus be given precedence.
Recognizable gaming	If some gaming is unavoidable, it would be best if it is possible to discern; metrics can be developed (having the same features as those listed above) to help recognize the gaming (e.g., excessive self-citations, hyperprolific authorship, citation orchestration).
Inclusion of indicators of best research practices	These could include data sharing, code sharing, protocol registration, and others.
Inclusion of indicators of poor research practices	These could include retractions, signs of image manipulations, paper mills, editorial nepotism practices, and others.

The potential value of centralized, science-wide, quantitative resources becomes even greater, if we also realize that qualitative assessments lead to many poor choices even in top institutions. Seemingly high-quality, but flawed, peer assessments then exert potent, negative influences on wider environments. The resignation of several leading scientists, including presidents and deans, from institutions such as Stanford and Cornell following documentation of problems with their research practices is probably just the tip of the iceberg.

Quantitative and qualitative assessments are not mutually exclusive. Even the most accurate quantitative tools are eventually interpreted by expert judgement. However, strengthening quantitative judgement in less-established institutions may be an affordable and realistic way towards approaching the ideal of parity among resource-wealthy and resource-poor institutions, empowering more the latter.

The endgame of any assessment is choices—excellent, good, or poor. Assessments operate like diagnostic tests. Excellent assessment tools and excellent assessors correctly “diagnose” (select) the best. Conversely, poor assessment tools and poor assessors miss the best scientists and exalt some of the worst. Admittedly, “excellence” is difficult to define and academic cultures that overuse the term may be rightfully criticized [14]. Moreover, rigorous, standardized, transparent, and reproducible quantitative metrics may not immediately abolish all inequities and corruption. They will nevertheless make many an unfairness obvious to the whole scientific community and perhaps even to the wider public, if what metrics mean, and their strengths and limitations are properly explained. Open, public documentation may put pressure on less-than-optimal systems to become more accountable.
